# Ultrasound Tongue Imaging in Research and Practice with People with Cleft Palate ± Cleft Lip

**DOI:** 10.1177/10556656231202448

**Published:** 2023-09-16

**Authors:** Joanne Cleland

**Affiliations:** 1 150865 Department of Psychological Sciences and Health, University of Strathclyde, Glasgow, UK

**Keywords:** ultrasound, articulation, biofeedback

## Abstract

Ultrasound tongue imaging is becoming popular as a tool for both phonetic research and biofeedback for treating speech sound disorders. Despite this, it has not yet been adopted into cleft palate ± cleft lip care. This paper explores why this might be the case by highlighting recent research in this area and exploring the advantages and disadvantages of using ultrasound in cleft palate ± cleft lip care. Research suggests that technological advances have largely overcome some of the difficulties of employing ultrasound with this population and we predict a future increase in the clinical application of the tool.

## Introduction

**Figure 1.**
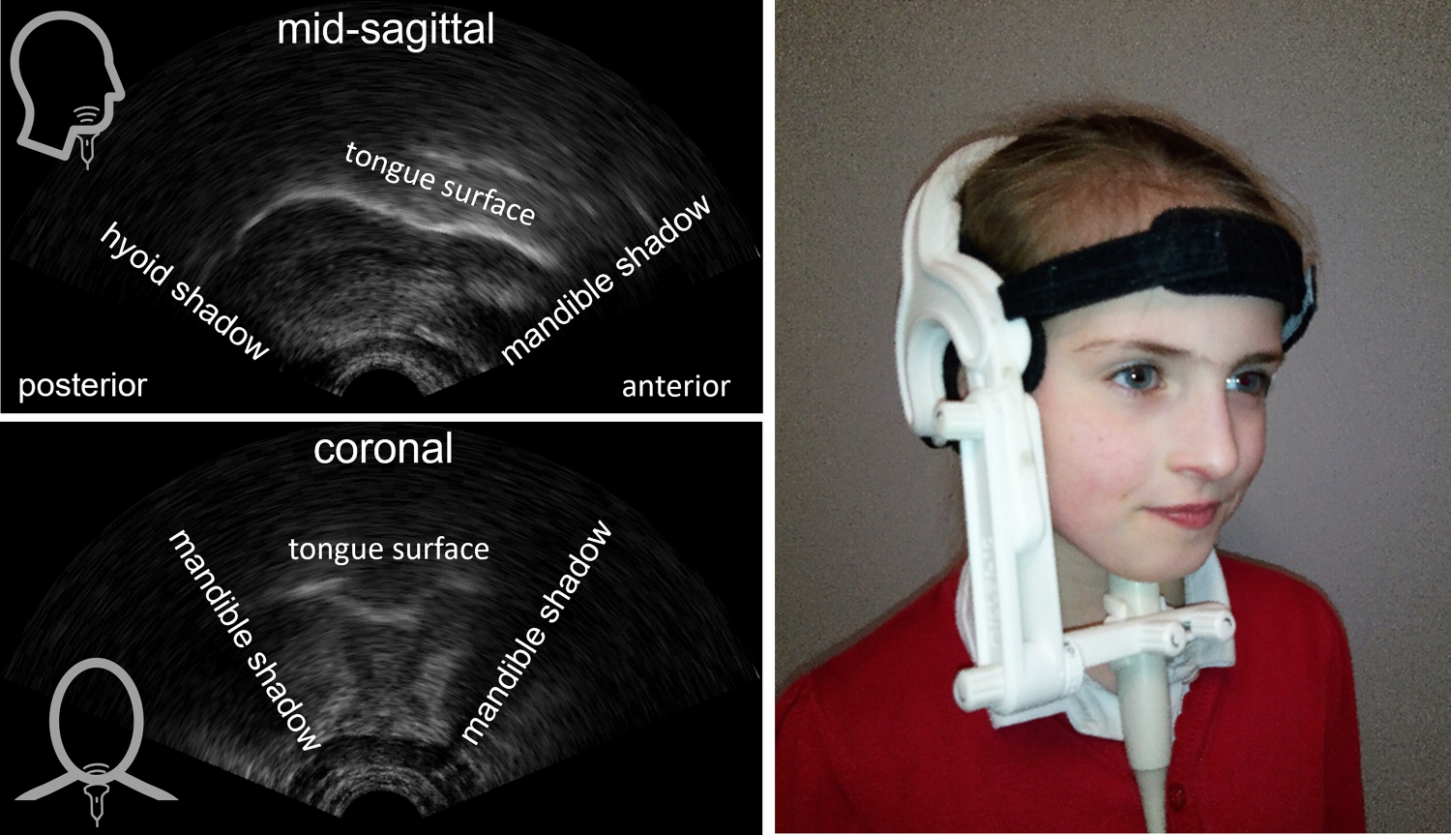
Ultrasound images of the tongue. Top left: mid-sagittal image with the tongue tip to the right. Bottom left: coronal image. In both images the brightest white line is the tongue surface. Right: Ultrafit headset with probe positioned for mid-sagittal view.

Speakers with cleft palate ± cleft lip (CP ± L) often have persistent difficulty with speech. Errors can be obligatory, for example, nasal realisation of voiced plosives, or compensatory, maladaptive articulatory placement to compensate for anatomical differences.^1^ Many of these compensatory errors affect lingual articulation, for example backing of alveolar consonants, either to other places within the oral cavity, such as velar/uvular or posterior to the oral cavity, for example glottal. Over the last 40 years there has been interest in measuring this lingual articulation instrumentally. Instrumental techniques can show us articulatory errors which are hard to identify and transcribe and (some) instrumental techniques can also be used in intervention. There are four main techniques for studying tongue movement: electropalatography (EPG), Magnetic Resonance Imaging (MRI), Electromagnetic Articulography (EMA), and Ultrasound Tongue Imaging (UTI).^2^ The technique of choice for speakers with CP  ±  L has historically been EPG.^3^ EPG displays tongue-palate contact^4^ but not tongue-shape. From EPG we have learnt that speakers with CP  ±  L show errors which we cannot always identify with phonetic transcription.^5^ For example, mid-dorsum palatal stops transcribed as velars; increased variability between repeated productions of the same consonant^6^; and covert contrasts in which two contrasting speech sounds such as /t/ and /k/ are perceived by a listener as identical, yet produced in subtly different ways.^7^ Identification of these errors can alter the type of treatment chosen. For example, a child with a covert contrast requires a motor-based articulatory intervention whereas a child with two consonants produced in an identical manner often needs a phonological approach. EPG can also be used to determine treatment targets^8^ and to treat compensatory errors when used as a biofeedback tool.^4^ Of the other techniques, MRI shows the entire vocal tract but it has not been used extensively for biofeedback because it is expensive, speakers must lie prone, affecting the position of the tongue root, and it is noisy, making acoustic recordings challenging. Similarly, EMA, which uses flesh-point tracking of (usually) three points on the tongue, is expensive and invasive and biofeedback applications are scarce in the literature. Ultrasound was first used for biofeedback, though not with CP  ±  L, in the 1980s. With this technique, an ultrasound probe is placed under the chin and either a mid-sagittal or coronal view of the tongue can be seen in real-time (Figure 1). Ultrasound is easy to use, non-invasive, and safe since it does not use ionising radiation.^9^ Despite this, its adoption into CP  ±  L research and practice, like most new tools,^10^ has been slow. Despite being in use since around the same time as EPG, historically EPG has won out as the tool of choice for CP  ±  L. Recent advances in ultrasound suggest this position is changing. Aside from the challenges of implementing change in healthcare systems, historically there were technical challenges with ultrasound: machines were cumbersome, expensive, had slow framerates, and ultrasound images were difficult to analyse. Most of these issues are now largely solved with the introduction of affordable compact systems with high framerates and analysis methods have seen significant improvements,^11^ and are set to improve further with advances in machine learning.^12^ Studies using ultrasound to treat speech sound disorders in children without CP  ±  L have rapidly increased^13^ and further studies are underway.^14,15^ A framework for the use of ultrasound in clinical practice is now available and is endorsed by the UK Royal College of Speech and Language Therapists^16^ and training in how to use ultrasound is available from universities, in an open access manual,^17^ and online at seeingspeech/speechstar.ac.uk.^18^ We predict that ultrasound will become an important tool for research and treatment in CP  ±  L, largely replacing other articulatory instruments as it has done for other types of speech sound disorder.^13^ Here we summarise what ultrasound can, and cannot, be used for by giving an overview of recent research specifically in CP  ±  L and conclude with future directions for this tool.

## Applications of Ultrasound Tongue Imaging

Mid-sagittal ultrasound shows almost the entire surface of the tongue from root to tip (Figure 1, left). Uvular and pharyngeal articulations, which occur in people with CP  ±  L,^19^ are clearly visible.^11^ Arguably, ultrasound is a far better tool for measuring retracted articulations than EPG or EMA as it can show post-velar articulations. This makes ultrasound ideal for treating compensatory errors involving backing of any type. In EMA the most posterior coil is usually attached to the back, not root, of the tongue^20^ and in EPG post-velar articulations are displayed as an ambiguous “open pattern” (ie, no tongue-palate contact).^6^ Bressmann et al.^21^ used ultrasound to identify that one speaker with CP  ±  L showed double articulations of a glottal plus pharyngeal articulation for /k/ which were auditorily perceived as only glottal stops. In the same paper, several speakers were shown to have mid-dorsum palatal stops in place of /k/. This demonstrates the use of ultrasound for identifying both covert contrasts and post-velar articulations. Identification of these errors can help clinicians target the precise error in intervention. Similarly, Cleland and colleagues^22^ developed a method for classifying lingual errors from ultrasound and demonstrated that in 39 children with CP  ±  L ultrasound could identify covert errors such as double articulations and retroflexion. When used in the mid-sagittal view, then, ultrasound is arguably the technique of choice for identifying differences in tongue shape and movement, helping to inform theoretical models and influence treatment plans. However, lateralised articulations, such as lateral fricatives, are common in CP  ±  L^6^ and these are not easily viewed with mid-sagittal ultrasound. In contrast, lateral fricatives are easily visualised and quantified in EPG because this technique shows tongue-palate contact across the entire palate. However, raising or lowering of the sides of the tongue can be visualised with coronal ultrasound^23,24^ (Figure 1). Nevertheless, this is problematic because it is very difficult to determine *which* coronal slice of the tongue is being imaged. This weakness of coronal ultrasound can be overcome using 3D ultrasound imaging,^25^ but so far this technique is cost-prohibitive and framerates are slow.

Another challenge with ultrasound has been analysis. While EPG consists of a normalised set of on/off contacts and EMA consists of a small, finite, number of sensors, ultrasound images are grainy and suffer from artefacts. Moreover, the image on the screen is not normalised and probe placement affects translation and rotation of the image. Because of these issues, most of the small number of studies using ultrasound with CP  ±  L have employed visual inspection methods.^21,22,26^ This method requires experienced clinical phoneticians viewing recordings of ultrasound videos and making judgments about tongue shape and movement. In a sense, this is like impressionistic phonetic transcription, but with an added visual modality. Cleland et al.^22^ showed that combining ultrasound and audio leads to better inter-transcriber agreement and identification of covert contrasts. This more accurate assessment method could lead to improved treatment plans, but this is yet to be tested. This qualitative method of evaluating ultrasound is similar to some EPG studies which describe and categorise EPG patterns^6^ and is quick and easy for clinicians wishing to use ultrasound in assessment before intervention.

## Quantifying Ultrasound

For ultrasound to be used to compare tongue shapes within and between speakers, either to measure changes post-intervention or to identify tongue shapes which differ from speakers without CP  ±  L, quantification is needed. In the phonetics literature of typical speech, ultrasound has become an increasingly important tool.^27^ This has led to several advancements in quantitative ultrasound methods including development of probe stabilising headsets^28^ (see Figure 1 for an example of a light weight headset) and systems which correct for head movement.^29^ Both of these make the ultrasound image easier to interpret and measurements more accurate. When the headset is used for biofeedback, it makes the image more stable for the client and clinician. Methods for tracking the surface of the tongue^30^; and machine learning for classifying speech errors^31^ show promise for developing automatic assessment tools. So far, quantitative ultrasound studies of CP  ±  L are scarce. Roxburgh and colleagues^32^ compared tongue contours statistically in covert contrasts in two speakers with CP  ±  L. They showed that ultrasound can be used to quantify the size of difference between phones produced with a covert contrast, and measure change during intervention. A further recent paper by Cleland et al.^33^ used the dorsum excursion index,^34^ to measure the relative excursion/height of the back of the tongue during production of high-pressure consonants. They hypothesised that children with CP  ±  L would show increased raising of the tongue back, due to an attempt to compensate for either current or resolved velopharyngeal insufficiency. They compared 31 children with CP  ±  L to 29 typically developing children. Although some individual children showed an unusually high and back tongue posture, they did not find group differences. They attribute this to the fact that many of the children with CP  ±  L in the group had normalised speech. Nevertheless, the study provides proof of concept that it is possible to use ultrasound metrics to compare groups of speakers with and without CP  ±  L and that it is possible to identify unusual raising of the tongue body with ultrasound, which in turn could be treated with ultrasound biofeedback.

## Biofeedback Applications

One of the main advantages of ultrasound is that it can be used in real-time, making it ideal as a biofeedback intervention. Biofeedback enables some speakers with persistent speech sound disorders to quickly correct articulations in one to two sessions.^35^ It therefore has the potential to improve the effectiveness and efficiency of articulation therapy, although children younger than age five or with additional learning needs may have difficulty understanding the ultrasound image.^8^ It has advantages over EPG in this application as it can be used without any individualised hardware and children do not need to have stable dentition. It is surprising, then, that this tool has not yet been widely adopted into cleft palate care. Two small-scale low-quality intervention studies, both with only two children, showed that ultrasound has potential as a biofeedback tool with CP  ±  L.^36,37^ The scarcity of literature in this area is surprising given that the number of studies has been increasing in other populations over the last 10 years.^13^ Possible reasons for the slow adoption in CP  ±  L include the fact that lateralisation errors are sometimes an intervention target^24^ but lowering and raising of the sides of the tongue can only be viewed in the coronal plane and this is technically difficult. However, backing errors including palatalisation of fricatives and backing to velar/uvular and pharyngeal, which are common, are ideal for treatment and slow adoption is likely due to the fact equipment costs have only just begun to become affordable in the last few years and it is only in the last year that a framework for clinical practice^16^ has been published. We predict that ultrasound will increasingly be adopted as a clinical tool, however, larger, more robust intervention studies specifically with CP  ±  L are clearly required alongside improvements in analysing ultrasound images automatically using machine learning approaches. One such study is currently underway in the UK,^14^ but further larger-scale studies will be needed.

## Conclusion

Ultrasound shows promise as an articulatory tool for research and practice with people with CP  ±  L. There is still much work to do on improving the affordability of the technology and streamlining analysis. Implementation of ultrasound into clinical practice will require more robust evidence of its effectiveness, and a greater understanding of the barriers to its use in speech and language therapy clinics, for example training needs and ongoing support needs. In the meantime, we encourage researchers and clinicians interested in adopting this technique to consult the increasing evidence base including that in the phonetics literature and the literature on other types of speech sound disorders, and the growing number of online resources such as www.seeingspeech.ac.uk/speechstar before applying this knowledge to CP  ±  L.
